# Patient-reported outcomes of lifestyle interventions in patients with severe mental illness: a systematic review and meta-analysis

**DOI:** 10.1186/s12888-022-03854-x

**Published:** 2022-04-13

**Authors:** Laura M. Pape, Marcel C. Adriaanse, Jelle Kol, Annemieke van Straten, Berno van Meijel

**Affiliations:** 1 Department of Health Sciences, Faculty of Science & Amsterdam Public Health Research Institute, Vrije Universiteit, Amsterdam, The Netherlands; 2 Department of Clinical, Neuro- and Developmental Psychology & Amsterdam Public Health Research Institute, Vrije Universiteit, Amsterdam, The Netherlands; 3 Department of Health, Sports & Welfare, Research Group Mental Health Nursing, Inholland University of Applied Sciences, Amsterdam, The Netherlands; 4 Amsterdam UMC (VUmc). Department of Psychiatry, Amsterdam Public Health Research Institute, Amsterdam, The Netherlands; 5 Parnassia Psychiatric Institute, Parnassia Academy, The Hague, The Netherlands

**Keywords:** Severe mental illness, Lifestyle intervention, Patient-reported outcome, Systematic review, Meta-analysis

## Abstract

**Background:**

Lifestyle interventions for severe mental illness (SMI) are known to have small to modest effect on physical health outcomes. Little attention has been given to patient-reported outcomes (PROs).

**Aim:**

To systematically review the use of PROs and their measures, and quantify the effects of lifestyle interventions in patients with SMI on these PROs.

**Methods:**

Five electronic databases were searched (PubMed/Medline, Embase, PsycINFO, CINAHL, and Web of Science) from inception until 12 November 2020 (PROSPERO: CRD42020212135). Randomised controlled trials (RCTs) evaluating the efficacy of lifestyle interventions focusing on healthy diet, physical activity, or both for patients with SMI were included. Outcomes of interest were PROs.

**Results:**

A total of 11.267 unique records were identified from the database search, 66 full-text articles were assessed, and 36 RCTs were included, of which 21 were suitable for meta-analyses. In total, 5.907 participants were included across studies. Lifestyle interventions had no significant effect on quality of life (*g* = 0.13; 95% CI = − 0.02 to 0.27), with high heterogeneity (*I*^*2*^ = 68.7%). We found a small effect on depression severity (*g* = 0.30, 95% CI = 0.00 to 0.58, *I*^*2*^ = 65.2%) and a moderate effect on anxiety severity (*g* = 0.56, 95% CI = 0.16 to 0.95, *I*^*2*^ = 0%).

**Discussion:**

This meta-analysis quantifies the effects of lifestyle interventions on PROs. Lifestyle interventions have no significant effect on quality of life, yet they could improve mental health outcomes such as depression and anxiety symptoms. Further use of patient-reported outcome measures in lifestyle research is recommended to fully capture the impact of lifestyle interventions.

**Supplementary Information:**

The online version contains supplementary material available at 10.1186/s12888-022-03854-x.

## Background

People with severe mental illness (SMI) have an increased risk of poor physical health and premature mortality. This can be attributed to the high prevalence of chronic somatic diseases in this patient group, including cardiometabolic diseases, respiratory diseases, and cancer [1–7]. Evidence suggests that people with SMI more often engage in risky health behaviours than the general population, including sedentary behaviour, low physical activity, unhealthy eating habits, smoking and substance abuse [8–11]. Given the severe health disparities, large efforts have been made to increase physical health among patients with SMI through behavioural interventions [6]. During the past decades, numerous studies on the efficacy of lifestyle interventions for patients with SMI have been executed [6, 12, 13].

Lifestyle interventions typically focus on weight management and aim to reduce overweight and obesity by stimulating dietary changes, decreasing sedentary behaviour, and increasing physical activity. However, recent systematic reviews and meta-analyses suggest that the effects of lifestyle interventions on physical health parameters, such as weight, body mass index (BMI), waist circumference, and blood pressure, are limited in this group [12], few show significant effects [13]. Especially interventions executed under real life conditions usually result in small to moderate effects that are oftentimes clinically insignificant [6, 12, 14]. Furthermore, to date there is limited information in long-term efficacy due to a lack of long-term follow-up studies [14]. This can lead researchers to be sceptical about the implementation of these interventions in clinical practice.

Little attention has been given to other possible benefits of lifestyle interventions such as improvements in quality of life (QoL), daily functioning, social functioning and participation, health-related well-being, or other patient-reported outcomes (PROs). PROs can be defined as ‘any report of the status of a patient’s health condition that comes directly from the patient, without interpretation of the patient’s response by a clinician or anyone else’ [15]. They are mostly self-report questionnaires but can also be acquired through interviews, diaries, or other tools [16]. PROs are valuable outcomes as they represent topics that are meaningful to patients and provide insight on the impact of interventions from the patient’s perspective [17, 18]. They often correlate poorly with objective physical outcomes or biomarkers, which emphasizes that a broad range of outcomes is needed to comprehensively capture the impact of lifestyle interventions [16]. Patients, health policy makers, and the scientific community have recognised the relevance of PROs, and their use in studies and clinical practice has increased in recent years [18–20]. However, the use of PROs in evaluation of lifestyle interventions has not been systematically evaluated and quantified yet.

The aim of this study is to systematically review the use of PROs and their patients-reported outcome measures (PROMs) in the evaluation of lifestyle interventions aiming at the promotion of healthy diet and physical activity for patients with SMI. We will furthermore quantify the effects of lifestyle interventions for SMI on three important PROs, which are quality of life, depression and anxiety.

## Method

### Search strategy and selection criteria

This systematic review was conducted according to the Preferred Reporting Items for Systematic Reviews and Meta-Analyses (PRISMA) guidelines [21] and it followed a beforehand published study protocol (PROSPERO registration number: CRD42020212135) [22]. Two researchers (LP and MA) developed and executed the search strategy with support of a mental health information specialist. The search was conducted in the databases PubMed/Medline, Embase, PsycINFO, CINAHL, and Web of Science from inception to 12 Nov 2020. We performed the search using search terms such as (“SMI” OR “severe mental illness*” OR “severe mental disorder*” OR “serious mental illness*” OR “serious mental disorder*”) AND (“life style” OR “health promotion” OR “physical fitness” OR “exercise” OR “healthy diet”) AND (“patient reported outcome measures” OR “prom”) AND “randomized controlled trial”). The full search string is shown in the Supplementary Material (Table S1). To identify any additional relevant studies, we systematically screened reference lists of key systematic reviews that were retrieved from the search string that was originally used as an orientation on currently available reviews on the topic.

We included randomised controlled trials (RCTs) only. Studies of all languages and publication dates were considered. We used the following four main domains of inclusion criteria to assess eligibility of the studies.

#### Participants

We included studies that included patients with SMI, using the definition of SMI by Delespaul and the consensus group SMI [23],stating that a psychiatric disorder can be defined as severe when the illness (1) requires coordinated treatment of health professionals; (2) is accompanied by serious limitations in social functioning; (3) is of chronic nature (structural or long-term, at least a few years) and not in symptomatic or functional remission; and (4) where the limitations are cause and consequence of the disorder [23]. Using these criteria, we included studies focusing on schizophrenia spectrum disorders or other psychotic disorders, bipolar disorder, severe personality disorder, or depressive disorder when chronicity was indicated. Studies with anxiety disorders, substance use disorders, eating disorders, or dementia as primary diagnosis were excluded.

#### Intervention

The included studies investigated lifestyle interventions focussing primarily on promoting physical activity, dietary changes, or a combination of both. We focussed on non-pharmacological interventions promoting weight loss, weight management, healthy diet, decrease of sedentary behaviour, or increase of physical activity.

#### Control condition

Studies with nonactive or minimally active control conditions were considered eligible (e.g. treatment as usual or waitlist control group).

#### Outcomes

We were interested in patient-reported outcomes (PROs), defined as ‘any report of the status of a patient’s health condition that comes directly from the patient without interpretation of the patient’s response by a clinician or anyone else’ [15], captured by self-report questionnaires, diaries, or other data collection tools [16].

### Data collection and analysis

#### Study selection

In the first round of selection, titles and abstracts were screened for eligibility using the Rayyan screening tool [24]. Literature was screened on the basis of our inclusion and exclusion criteria by the first author (LP). At the start, two other researchers (MA and BvM) independently screened a smaller sample of each 5% of all records (*n* = 1.145). Selection criteria were defined in greater detail which ultimately led to consensus. Additionally, a selection of articles that were cases of doubt (*n* = 160) and were screened by only one researcher (LP) in the first round. These underwent a second screening by two researchers for a definite decision (LP and MA). Disagreements in inclusion and exclusion were resolved by discussion. Disagreements or uncertainties were discussed with the senior researcher (BvM).

**Fig. 1:**
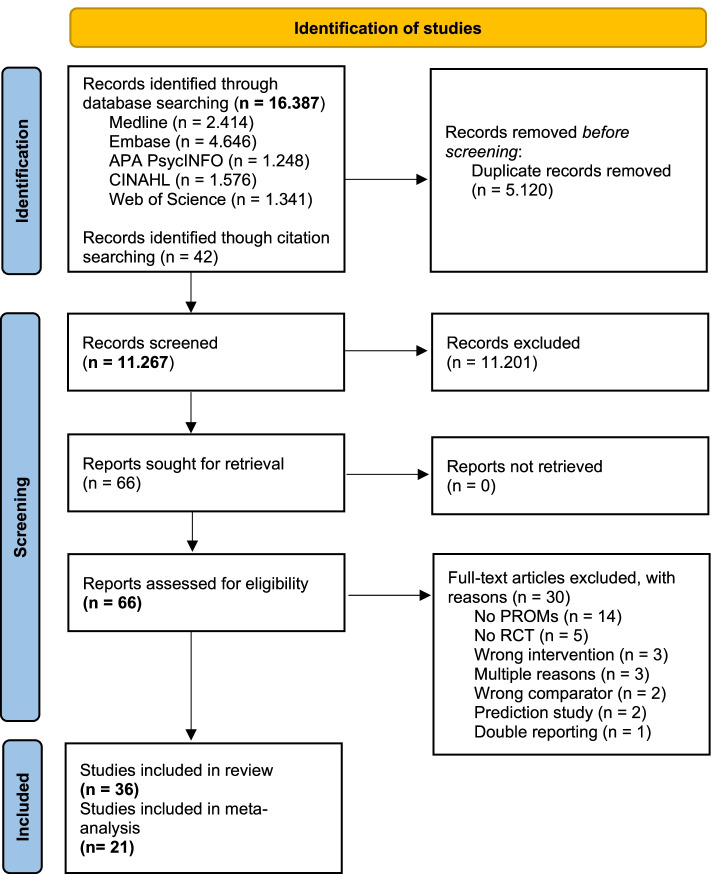
PRISMA flow diagram of study search and selection

In the second round of screening, each full-text article was screened independently by two researchers (LP and JK). Disagreements were resolved by discussion or decision by a third and fourth researcher (MA and BvM). An overview of the study selection process can be found in the PRISMA flow diagram (Fig. 1).

#### Data extraction

The process of data extraction was carried out by two persons independently (LP and JK). The data was extracted using a standardised data extraction file which was developed beforehand. The following items were extracted for description of study characteristics: first author, year of publication, country, setting and diagnosis, sample size, mean age, intervention (intervention aim, focus, format, components, duration, and delivery), control group, follow-up moments and PROM questionnaires. Additionally, data for quality assessment and meta-analysis was extracted, and risk of bias assessment was done by two independent researchers (JK and LP). Discrepancies were once again resolved by discussion.

#### Risk of bias assessment

The Cochrane Risk of Bias Tool 2.0 was used to assess the methodological limitations of the included studies [25]. Risk of bias assessment was performed independently by two researchers (LP and JK). The following domains were assessed: (1a) the randomisation process; (1b) identification or recruitment of participants into clusters; (2) deviations from intended interventions; (3) missing outcome data; (4) measurement of the outcome; and (5) selection of the reported result [25]. The risk of bias for each domain was scored as either low, high, or with some concern, and an overall judgement for each study was made. In addition, we made a distinction between high-risk studies and ‘lower-risk’ studies. The fourth domain was removed for this purpose, as it was expected to score as ‘high risk’ in any case because of the inability of blinding in lifestyle intervention trials. Studies were labelled ‘lower risk of bias’ when at least three of the remaining domains scored low risk and none of the domains scored high risk.

#### Quality assessment

The general quality of the evidence was assessed (LP) using the Grading of Recommendations Assessment, Development, and Evaluation (GRADE). Five GRADE domains were assessed: (1) risk of bias, (2) imprecision, (3) indirectness, (4) heterogeneity, and (5) publication bias. Possible ratings for each meta-analysis were either high, moderate, low, or very low, representing the strength of the evidence [26].

#### Outcome measures

The outcomes of interest were PROs [15]. The conceptual model of Wilson & Cleary was used to provide the theoretical framework [27]. The model divides outcomes into five categories: biological and physiological variables, symptom status, functional status, general health perceptions, and overall QoL. We considered the model while analysing the concepts of the different PROs and in deciding which ones should be pooled in the meta-analysis. For the meta-analysis, we chose the most frequently used PROMs that measured the health status of a patient rather than health behaviour, as we considered those as most relevant and meaningful for patients. Based on these criteria, quality of life, depression severity, and anxiety severity were considered the most important outcomes.

#### Data synthesis and statistical analysis

For the meta-analyses, we used widely accepted PROMs. The decisions on which PROMs were similar enough to be pooled in meta-analysis was made based on the underlying construct and items of each PROM [16]. We used the means, standard deviations, and sample size of each intervention and control group, or alternatively the *p*-values and sample sizes to calculate the effect size. When more than one outcome of the same construct was reported in one study, we performed a sensitivity analysis, pooling an effect size for the lowest effect sizes, the highest effect sizes, and all effects combined. Studies were considered outliers if their 95% confidence interval (CI) lied outside of the 95% CI of the pooled effect. Meta-analysis was conducted for the outcomes quality of life, depression, and anxiety. The Comprehensive Meta-analysis software (Version 3.3.070) was used to calculate the Hedges’ g statistic with 95% confidence intervals (CI) using the random effects model (www.meta-analysis.com). In this context, a Hedges’ g of 0.2 would be considered as minor, 0.5 as moderate, and 0.8 as a major effect [28].

Heterogeneity was assessed using the *I*^*2*^-statistic, with scores of < 25%, 25-­50 and > 50%, indicating low, moderate, and high heterogeneity, respectively [29]. We examined the heterogeneity and differences in effect sizes of specific groups by executing subgroup analyses and exploratory analyses using the mixed-effects analysis. Publication bias was assessed graphically by inspecting funnel plots and statistically by utilizing Egger’s regression tests [29].

## Results

### Study selection

After removal of duplicates, a total of 11.267 records were obtained from the databases. By applying the predefined eligibility criteria, we selected 66 records for full-text screening. Thirty articles failed to meet the inclusion criteria and were subsequently excluded. We included 36 studies meeting the inclusion criteria. Twenty-one studies were included in the meta-analyses. Fourteen studies could not be pooled, as they included PROMs that were not reported frequently enough (e.g. self-esteem or loneliness), or only included PROMs focussing on health behaviour (e.g. registration of dietary behaviour or physical activity). One study did not provide sufficient data for the analysis of quality of life in terms of missing sample size per condition and effect size data [30]. Details on the study selection process can be found in Fig. 1.

### Study characteristics

Table 1 shows a summary of the key characteristics of all 36 included RCTs. We included studies from 15 different countries of which 47% European (*n* = 17) [30, 35–37, 39, 42–44, 47–49, 51, 53, 54, 56, 61, 62], 31% North American (*n* = 11) [33, 34, 38, 40, 45, 52, 55, 57, 60, 63, 65], 8% Asian (*n* = 3) [46, 50, 58], 8% Australian (*n* = 3) [32, 41, 64], and 6% South American origin (*n* = 2) [31, 59]. At baseline, a total of 5.907 participants were enrolled across studies. The studies were published from 2005 until 2020 and 56% (*n* = 20) were published during the past 5 years. The studies had a sample size ranging from 13 to 814 participants (mean/median = 164/101). The mean age of the participants ranged from 31 to 60 years. The percentage of male participants ranged from 14 to 100% (mean/median = 56/52). The main primary diagnoses were schizophrenia spectrum disorders or psychotic disorders in 86% of the included trials (*n* = 32). Other primary diagnoses were bipolar disorder (*n* = 2) and major depressive disorder (*n* = 2). Participants were recruited from outpatient settings in 86% of all trials (*n* = 31), in some trials from inpatient clinics (*n* = 4), or a combination of both (*n* = 1).

**Table 1:** List of all included studies – Study characteristics

Author (Year) Country	Sample characteristics			Intervention	Control	Follow-up	PROs & PROMs	Findings^**a**^
	Setting & Diagnosis	Sample size	Mean age (SD)					
Attux et al. (2013) [31] Brazil	Clinically stable outpatients with schizophrenia spectrum disorder	160	IG 36.2 (*SD* 9.9); CG 38.3 (*SD* 10.7)	12-week Lifestyle Wellness Program including one-hour weekly sessions to discuss topics like dietary choices, lifestyle, physical activity and self-esteem with patients and their relatives	TAU	0 M 3 M 6 M	**QoL** (World Health Organization Quality of Life Questionnaire (WHO-QOL-BREF)) **Self-esteem** (Rosenberg Self-esteem scale) **Living skills** (Independent Living Skills Survey (ILSS)) **Dietary fat and fibre intake** (Dietary, Instrument for Nutrition Education (DINE)) **Physical activity** (International Physical Activity Questionnaire (International Physical Activity Questionnaire (IPAQ)) **Smoking** (Fagerström tolerance questionnaire)	No significant differences between groups in PROs
Baker et al. (2015) [32] Australia	Smoking outpatients with schizophrenia spectrum or bipolar disorder, or other psychotic disorders	235	41.6 (*SD* 11.1)	9-month face-to-face lifestyle intervention including one 90-min session and a total of 16 one-hour sessions to discuss topics like smoking cessation and other CVD risk behaviours such as physical activity and healthy eating habits	Telephone-based intervention with discussions on comparable topics, but less intensive	0 W 15 W 12 M	**HRQoL** (12 item Short Form survey (SF-12) **QoL** Impact of Weight on Quality Of Life (IWOQOL-lite)) **Depression symptoms** (Beck Depression Inventory (BDI-II) **Weekly activity in walking and sitting** (IPAQ) **Daily servings of vegetables, fruit, or combined** (24 h eating habits recall) **Smoking** (Fagerström test for nicotine dependence; Opiate Treatment Index (OTI); Self-reported cigarettes per day; Smoking abstinence) **Readiness and Motivation to quit smoking** (RQM) **Alcohol and cannabis use** (OTI)	No significant differences between groups in PROs
Bartels et al. (2013) [33] USA	Outpatients with schizophrenia spectrum, bipolar or major depressive disorder	133	43.8 (*SD* 11.5)	12-month In SHAPE Lifestyle intervention including weekly one-hour sessions with a fitness trainer with combined nutrition and health education plus a fitness club membership	One year of fitness club membership and education	0 M 3 M 6 M 9 M 12 M	**Physical activity** (International Physical Activity Questionnaire - Short-form (IPAQ-SF)), **Readiness to change dietary behaviour** (Weight Loss Behaviour–Stage of Change Scale (WLB-SOC))	Significant differences between groups in improvements in readiness to engage in nutrition behaviours (WLB-SOC), minutes exercised per week, total vigorous activity score (IPAQ) at 12-month follow-up
Bartels et al. (2015) [34] USA	Outpatients with schizophrenia spectrum, bipolar or major depressive disorder	210	43.9 (*SD* 11.2)	12-month In SHAPE Lifestyle intervention including weekly one-hour sessions with a fitness trainer with combined nutrition and health education plus a fitness club membership	One year of fitness club membership and education	0 M 3 M 6 M 9 M 12 M 18 M	**Physical activity** (International Physical Activity Questionnaire - Short-form (IPAQ)) **Readiness to change dietary behaviour** (Weight Loss Behaviour–Stage of Change Scale (WLB-SOC)) **Dietary behaviour** (Brief Block Food Frequency Questionnaire (FFQ))	Significant differences between groups in improvements in readiness to change nutrition behaviours (WLB-SOC), exercise minutes and total vigorous activity score (IPAQ) at 12-month follow-up, maintenance of effects at 18-months with exception of decreases in WLB-SOC in IG relative to CG
Battaglia et al. (2013) [35] Italy	Outpatients on a stable antipsychotic pharmacological program with schizophrenia spectrum disorder	18	IG 36.0 (*SD* 5.0); CG 35.0 (*SD* 4.0)	12-week exercise intervention program including two-hour soccer training sessions twice a week	TAU	0 W 12 W	**HRQoL** (Short Form Health Survey (SF-12))	Significant differences between groups in improvements in the physical and mental domain of the SF-12 scores at 12-week follow-up
Bersani et al. (2017) [36] Italy	Clinically stable inpatients with schizophrenia spectrum, bipolar or major depressive disorder	32	IG 52.6 (*SD* 13.7); CG 52.0 (*SD* 13.4)	5-week psychoeducational intervention including 90-min weekly sessions discussing topics like sleep, physical activity, diet and the consequences of voluptuary habits	Five psycho- educational group sessions discussing clinical outcomes or watching and discussing movies on pharmacological therapy	0 W 5 W	**Sleep quality** (Pittsburgh Sleep Quality Index (PSQUI)), **Physical activity** (International Physical Activity Questionnaire (IPAQ)), **Adherence to the Mediterranean Diet** (QUMDA)	Significant differences between groups in improvements in sleep quality (PSQUI) and adherence to diet (QUMDA) at 5-week follow-up
Bonfioli et al. (2018) [37] Italy	Outpatients in community psychiatric services with affective or non-affective functional psychosis disorders	325	IG 44.6 (*SD* 10.3); CG 47.5 (*SD* 10.8)	6-month intervention program including seven one-hour health education group sessions discussing physical activity and diet, weekly one-hour group walking sessions and regular calls promoting adherence	TAU	0 M 6 M	**Physical activity and dietary habits** (Progressi delle Aziende Sanitarie per la Salute in Italia (PASSI))	Significant differences in improvements between groups in physical activity (PASSI) at 6-month follow-up
Brar et al. (2005) [38] USA	Clinically stable long-term inpatients and outpatients with schizophrenia spectrum disorder	71	IG 40.0 (*SD* 10.1); CG 40.5 (*SD* 10.6)	14-week behavioural treatment intervention including 20 sessions teaching behavioural techniques for weight loss	TAU	0 W 4 W 8 W 14 W End-point	**Client satisfaction** (Client Satisfaction Questionnaire (CSQ-8))	Significant differences in improvements between groups in client satisfaction (CSQ-8) at endpoint (endpoint unclear)
Brown & Chan [39] (2006) UK	Outpatients from a community mental health team with primary ICD-10 diagnosis of psychosis, major affective illness or severe personality disorder	28	IG 45.1; CG 41.7	6-week lifestyle intervention including weekly, 50 min, one-to-one health promotion sessions discussing topics like weight control, healthy eating, exercise, structured daily activity and substance misuse	Waiting-list (TAU during study period)	0 W 6 W	**Dietary habits** (Dietary Instrument for Nutrition Education (DINE)) **Physical activity** (GODIN questionnaire) **Psychological health** (Hospital Anxiety and Depression scale (HAD)) **Self-rated physical health, physical fitness and mental health** (Self-reported Likert scale) **Alcohol use** (Not specified) **Smoking** (Not specified)	Significant differences between groups in improvements in moderate exercise (GODIN) at 6-week follow-up
Erickson et al. (2006) [40] USA	Outpatients with schizophrenia spectrum, bipolar or posttraumatic stress disorder with psychotic symptoms	122	IG 49.7 (*SD* 6.9); CG 49.6 (*SD* 9.1)	12-month Lifestyle Balance behavioural intervention program including weekly classes and individual counselling for 8 weeks, food and exercise diaries, rewards, caregiver consultations, and monthly booster classes and counselling	TAU provided with self-help materials for weight loss, exercise and nutrition	0 M 2 M 6 M 12 M	**Insight** (Self-appraisal of Illness Questionnaire (SAIQ)) **Motivation** (Motivational Interview to Assess Stage of Change (MI))	No significant differences between groups in PROs
Evans et al. (2005) [41] Australia	Outpatients with schizophrenia spectrum, bipolar or major depressive disorder	51	IG 34.6 (*SD* 9.6); CG 33.6 (*SD* 11.6)	3-month nutritional intervention program to prevent weight gain including six one-hour education sessions discussing dietary components and physical activity	TAU (passive nutritional education from the booklet)	0 M 3 M 6 M	**QoL** (Not specified) **Overall health** **Body image** **Activity level** (All self-report scales based on Clinical Global Impressions (CGI))	Significant differences between groups in improvements in subjective quality of life, overall health, body image, and activity level (CGI) at 3-month follow-up
Fernandez Guijarro et al. (2019) [42] Spain	Outpatients from community mental health centers with schizophrenia spectrum, bipolar or major depressive disorder	61	47.0 (*SD* 9.2)	24-week nurse-led lifestyle modification program including weekly group sessions discussing lifestyle-related topics and booklets with information on various lifestyle topics	TAU	0 M 6 M	**Overall health status** (Euro-QoL (EQ-5D)) **Smoking** Fagerström tolerance questionnaire **Physical activity and sitting time** (International Physical Activity Questionnaire – Short Form (IPAQ-SF))	Significant differences between groups in improvements in physical activity and sitting time (IPAQ-SF) and overall health (EQ-5D) at 6-month follow-up
Forsberg et al. (2010) [43] Sweden	Patients living in supported housing with schizophrenia, bipolar disorder or other psychotic or psychiatric disorders	41	IG 39.8 (*Range* 23-59); CG 42.8 (*Range* 22-71)	12-month health intervention programme consisting of two-hour study circles twice a week, once a week for diet sessions and once a week for physical activities	Aesthetic study circle to learn and practice artistic techniques	0 M 12 M	**HRQoL** (Study 36 Item Short Form (SF-36)) **QoL** (The Manchester Short Assessment of Quality of Life (MANSA)) **Psychiatric symptoms** (Symptom Check List (SCL-90-R)) **Sense of coherence** (SOC-scale)	Significant differences between groups in improvements in sense of coherence (SOC scale) between groups at 12-month follow-up
Gaughran et al. (2017) [44] UK	Outpatients from community mental health teams with established psychotic disorder	406	44.2 (*SD* 10.12)	9-month IMPACT lifestyle intervention including 30-min sessions using motivational interviewing techniques to address lifestyle choices, with modules targeting key lifestyle components	TAU	0 M 12 M 15 M	**HRQoL** (36-Item Short Form Health Survey (SF-36)) **Smoking** (Fagerström Nicotine Dependence Questionnaire) **Alcohol use** (Alcohol Use Disorders Identification Test (AUDIT)) **Cannabis and illegal substance use** (Time Line Follow Back) **Dietary habits** (Dietary Instrument for Nutrition Education (DINE)) **Physical activity** (International Physical Activity Questionnaire – Short Form (IPAQ-SF))	No significant differences between groups in PROs
Goldberg et al. (2013) [45] USA	Veterans from outpatient mental health clinics with schizophrenia spectrum, bipolar disorder, major depressive, or severe anxiety disorder	109	52.0 (*SD* 9.1)	6-month MOVE! intervention program including psychoeducation on dietary components, with weekly 60-min sessions for the first four months, followed by four biweekly sessions, and two individual sessions	TAU (plus basic information about diet and exercise every month)	0 M 6 M	**HRQoL** (12-Item Short Form (SF-12)) **QoL** Impact of Weight on Quality of Life Survey (IWQOL)) **Dietary habits** (Block Fruit, Vegetable, and Dietary Fat Screeners) **Physical activity** (Not-specified) **Attitude and motivation** (Diet and Exercise Confidence Survey)	No significant differences between groups in PROs
Ho et al. (2016) [46] China	Inpatients residing in a mental health rehabilitation hostel with chronic schizophrenia	153	54.0 (*SD* 8.4)	3-month intervention program including weekly 60-min exercise classes for 12 consecutive weeks and twice-weekly 45-min practice sessions (IG1)	IG2 Tai-Chi intervention; CG Waiting-list (TAU during study period)	0 M 3 M 6 M	**Perceived stress** (Chinese perceived stress scale (PSS)) **Daily functioning** (Chinese version of the Barthel’s Activities of Daily Living (ADL) index; Lawton’s Instrumental Activities of Daily Living Scale (IADL))	Significant improvements in daily functioning (ADL) in IG1 compared to CG at 3 months
Holt et al. (2019) [47] UK	Outpatients in community mental health trusts with schizophrenia spectrum disorder or first episode psychosis	414	IG 40.0 (*SD* 11.3); CG 40.1 (*SD* 11.5)	12-month STEPWISE intervention aimed at weight loss including four weekly 2.5-h group sessions discussing dietary and physical activity components complemented with three booster sessions and individual support contact	TAU with printed advice on lifestyle and risks associated with weight gain	0 M 3 M 12 M	**Dietary intake** (Adapted Dietary Instrument for Nutrition Education questionnaire (DINE)) **HRQoL** (RAND SF-36) **QoL** (EQ-5D-5L) **Health beliefs** (Adapted Brief Illness Perception Questionnaire) **Depressive symptoms** (9-item Patient Health Questionnaire (PHQ-9)) **Smoking status** (Not specified)	No significant differences between groups in PROs
Jakobsen et al. (2017) [48] Denmark	Outpatients with schizophrenia spectrum disorder or persistent-delusional disorder	428	38.6 (*SD* 12.4)	12-month CHANGE lifestyle program including lifestyle coaching consisting of weekly one-hour individual meetings supporting and motivating physical activity, healthy dietary choices and smoking cessation (IG1)	IG2 Care coordination; CG TAU	0 M 12 M 24 M	**QoL** (Manchester Short Assessment of Quality of Life (MANSA)) **Physical activity** (Physical Activity Scale (PAS)) **Smoking** (Fagerström Test for Nicotine Dependence) **Dietary habits** (24-h recall interview & Food Frequency Questionnaire) **Perceived health** (Likert scale)	No significant differences between groups in PROs
Kaltsatou et al. (2015) [49] Greece	Inpatients with schizophrenia recruited from psychiatric outpatient department	31	59.9 (*SD* 14.1)	8-month supervised exercise training programme with Greek traditional dancing, 3 times/week	TAU (sedentary control)	0 M 8 M	**QoL** (Quality of Life Enjoyment and Satisfaction Questionnaire (Q-LES-Q))	Significant differences between groups in improvement in overall QoL and subscales physical health, subjective feelings, household duties, leisure activities, social relationships and general activities at 8-month follow-up
Kwon et al. (2006) [50] South Korea	Outpatients from clinical centres with schizophrenia or schizoaffective disorder	48	IG 32.0 (*SD* 9.2); CG 29.8 (*SD* 6.1)	12-week weight management program based on diet and exercise management, once/week and after week 4 every other week	TAU with verbal recom-mendations as to their physical activity and eating behaviour	0 W 4 W 8 W 12 W	**QoL** (World Health Organization Quality of Life (WHO-QOL-BREF)) **Eating inventory** (Not specified)	No significant differences between groups in PROs
Looijmans et al. (2019) [51] Netherlands	SMI patients from community-care and sheltered-living teams with psychotic disorders, mood disorders, personality disorders or anxiety disorders	244	46.1 (*SD* 10.8)	12-month multimodal, patient-centred lifestyle intervention to improve patients’ cardio-metabolic health delivered by mental health nurses, once every two weeks	TAU (Routine Outcome Monitoring assessment)	0 M 6 M 12 M	**Patient readiness to change physical activity** **Patient readiness to change dietary behaviour** (Both stages of change model, 5 point Likert-scale)	Significant differences between groups in improvement of readiness to change eating behaviour at 6- and 12- month follow-up, but none in readiness to change PA
Marzolini et al. (2009) [52] Canada	Patients from an Assertive Community Treatment (ACT) team with severe schizophrenia/ schizoaffective disorder	13	44.6 (*SD* 3.0)	12-week, community-based, group exercise program of either aerobic training twice/week (IG1)	IG2 resistance training; CG TAU	0 W 12 W	**Emotional functioning** (Mental Health Inventory (MHI))	No significant differences between groups in PROs
Masa-Font et al. (2015) [53] Spain	Outpatients from public mental health teams with schizophrenic, schizoaffective or bipolar disorder	332	IG 46.3 (*SD* 8.9); CG 47.1 (*SD* 9.9)	3-month physical activity and diet educational group program, twice/week	TAU	0 M 3 M	**HRQoL** (36-Item Short Form Health Survey (SF-36)) **Physical Activity** (International Physical Activity Questionnaire (IPAQ)) **Dietary habits** (Mediterranean Diet Assessment Tool (PREDIMED)	Significant differences between groups in improvement of physical activity and in the physical component of SF-36 at 3-month follow-up
Mauri et al. (2008) [30] Italy	Outpatients with bipolar disorder, schizoaffective disorder, or psychotic depression	49	38.9 (*range* 19-60 years)	24-week psychoeducational program (PEP) for weight loss based on a dietary program, eight monthly meetings total	No intervention, but continuing olanzapine, after 12 weeks starting PEP	0 W 12 W 24 W	**QoL** (Quality of Life Enjoyment and Satisfaction Questionnaire (Q-LES-Q-SF))	No significant differences between groups in PROs
McCreadie et al. (2005) [54] Scotland	Schizophrenic patients living on their own or in supported accommodations	102	45 (*SD* 13)	6 months of free fruit and vegetables supported by instruction in meal planning and food preparation (IG1) or free fruit and vegetables alone (IG2)	TAU	0 M 6 M 12 M 18 M	**Number of portions of fruit and vegetables eaten per week** (Scottish Health Survey Questionnaire)	Significant improvements in fruit and vegetable intake in both IGs compared to CG after 6 months, decrease in consumption back to baseline in IG2 and more gradually in IG1 after 12 months
McKibbin et al. (2006) [55] USA	Patients from board-and-care facilities and day treatment programs with schizophrenia or schizoaffective disorder	64	IG 53.1 (*SD* 10.4); CG 54.8 (*SD* 8.2)	24-week Diabetes Awareness and Rehabilitation Training, weekly sessions addressing diabetes education, nutrition, and lifestyle exercise	TAU plus brochures from the American Diabetes Association relevant to diabetes management	0 M 6 M	**Diabetes self-efficacy** (Diabetes Empowerment Scale) **Dietary habits** (Block Brief 2000 Revision of the Health and Habits and History Questionnaire) **Physical activity** (Yale Physical Activity Scale (YPA))	Significant differences between groups in improvements in diabetes self-efficacy, physical activity, and reductions in fat consumption and trend toward greater percentage of calories derived from protein sources at 6 months follow-up
Mota-Pereira et al. (2011) [56] Portugal	Outpatients with treatment-resistant non-remitted Major Depressive Disorder	33	IG 48.7 (*SD* 2.3); CG 45.3 (*SD* 3.1)	12-week exercise program of moderate home-based walks, five times/week, once per week supervised plus usual pharmacotherapy	TAU (usual pharma-cotherapy)	0 W 4 W 8 W 12 W	**Depression severity** (Beck Depression Inventory (BDI-II))	Significant differences between groups in improvements in depression severity (BDI-II) at 12 week follow-up
Muralidharan et al. (2020) [57] USA	Outpatients with schizophrenia, schizoaffective disorder, affective psychoses, post-traumatic stress disorder	276	IG1 53.7 (*SD* 9.6); IG2 54.7 (*SD* 8.9); CG 54.2 (*SD* 9.9)	6-month in-person ‘MOVE’ weight management intervention, 24 group and/or individual sessions including psychoeducation, goal-setting, and weekly weigh-ins (IG1)	IG2 Online-delivered ‘MOVE’; CG TAU	0 M 3 M 6 M	**Mental health treatment outcomes** (Revised Behaviour and Symptom Identification Scale (BASIS-R)) **Loneliness** (Three-Item Loneliness Scale) **QoL** (Lehman Quality of Life Interview - Brief Version; Impact of Weight on Quality of Life (IWQOL-Lite)) **HRQoL** (Veterans RAND 12 Item Health Survey (VR-12))	Significant improvements in loneliness and mental health related quality of life at 6 months at IG1 (in-person); significant improvements in mental health-related quality of life at 3 and 6 months, and in weight-related self-esteem at 6 months in IG2 (web-based)
Ryu et al. (2020) [58] South Korea	Outpatients from psychiatric units and community mental health centres with schizophrenia or schizoaffective disorder	60	IG 38.7 (*SD* 10.1); CG 39.0 (*SD* 8.6)	16-week group-based supervised and structured outdoor cycling program, 90 min/week	Occupational therapy	0 W 4 W 8 W 12 W 16 W	**Depression severity** (Beck Depression Inventory (BDI)) **State anxiety** (State and Trait Anxiety Inventory (STAI)) **Self-esteem** (Rosenberg Self-Esteem Scale (RSES)) **QoL** (World Health Organization Quality of Life (WHOQOL-BREF)) **Physical Activity** (Physical Activity Scale for the Elderly – Korean version (K-PASE))	Significant differences between group improvement of state and trait anxiety levels (STAI), and depressive symptoms (BDI) at 16 week follow-up
Silva et al. (2015) [59] Brazil	Patients from mental health clinics with schizophrenia	47	IG1 33.4 (*SD* 12.2); IG2 32.9 (*SD* 2.3); CG 33.6 (*SD* 2.6)	20-week program of concurrent exercise twice a week (IG1)	IG2 resistance exercise; CG Occupational therapy	0 W 10 W 20 W	**HRQoL** (36-Item Short Form Health Survey (SF-36))	Significant improvements in the role-physical domain of SF-36 in both IGs compared to CG at 20 week follow-up
Skirnar et al. (2005) [60] USA	Patients from inpatient, partial hospitalization and outpatient units or community treatment centres with psychotic or mood disorders	30	IG 39.7 (*SD* 8.2); CG 36.3 (*SD* 11.3)	12-week healthy lifestyle and fitness intervention, exercise session four times/ week and health seminars once/week	Waiting-list (TAU during study period)	0 W 12 W	**Psychiatric symptoms** (Symptom Checklist-90-R (SCL-90-R)) **QoL** (Lehman Quality of Life Questionnaire; SF-36) **Self-efficacy/ Empowerment** (Boston University Making Decisions Questionnaire)	Significant differences in improvements of general health (Lehman questionnaire) and self-efficacy (Boston University questionnaire) at 12 week follow-up
Speyer et al. (2016) [61] Denmark	Outpatients with schizophrenia, schizoaffective disorder or persistent delusional disorder	428	38.6 (*SD* 12.4)	12-month CHANGE lifestyle coaching plus care coordination plus treatment as usual (IG1)	IG2 Care coordination only; CG TAU	0 M 12 M	**Physical activity** (Physical Activity Scale (PAS)) **Smoking** (Fagerström Test for Nicotine Dependence) **Dietary habits** (Dietary Quality Score) **QoL** (EQ-5D; Manchester Short Assessment of Quality of Life (MANSA)) **Perceived health** (Not specified) **Perceived stress** (Perceived Stress Scale (PSS))	No significant differences between groups in PROs
Stiekema et al. (2018) [62] Netherlands	Patients in sheltered housing or clinical care facilities with psychotic disorders, personality disorder or mood disorders	814	48.6 (*SD* 12.5)	12-month ELIPS diet-and-exercise lifestyle intervention targeting the obesogenic environment, several times intensive contact and activities during first 3 months, followed by 9 months monitoring phase	TAU	0 M 3 M 12 M	**QoL** (Manchester Short Assessment of Quality of Life (MANSA))	Significant reduction in quality of life (overall) in the intervention group after 3 and 12 months follow-up (between group differences MANSA in favour of the CG)
Sylvia et al. (2019) [63] USA	Patients with bipolar disorder type I or II	38	42.0 (*SD* 12.3)	20-week ‘NEW Tx’ intervention, integrated CBT-based lifestyle intervention CBT-based with main modules nutrition, exercise, and wellness, weekly sessions (18 total)	Waiting-list (TAU during study period)	0 W 10 W 20 W	**Physical activity** (Exercise Questionnaire (EQ))	No significant differences between groups in PROs
Usher et al. (2013) [64] Australia	Outpatients from local mental health services including NGOs with schizophrenia, bipolar disorder, or other psychotic disorders	101	NA.	12-week nurse-led weight management and exercise intervention, weekly sessions of health education and group physical activity	12-week healthy lifestyle booklet	0 W 12 W	**Subjective effects of neuroleptic medications** (Drug Attitude Inquiry-10 (DAI-10); Liverpool University Neuroleptic Side Effect Rating Scale) **HRQoL** (Medical Outcomes Study Short Form 36 (SF-36))	No significant differences between groups in PROs
Yarborough et al. (2016) [65] USA	Outpatients in community mental health centres with schizophrenia spectrum disorders	200	47.2 (*SD* 10.6)	12-month STRIDE Weight Loss and Lifestyle Intervention, 6 month weekly group meetings on nutrition and PA, 6 months monthly meetings maintenance phase	TAU	0 M 6 M 12 M 24 M	**Body Image** (Body Weight, Image and Self-Esteem Evaluation questionnaire (B-WISE)) **General health** (SF-36v2 general health subscale) **Health related self-efficacy** (Patient Activation Measure (PAM))	Significant differences between groups in improvements in body image (B-WISE) at 12 and 24 months, and for general health (SF-36) and health related self-efficacy (PAM) at 24 months follow-up

*Abbreviations*: *IG* Intervention group, *CG* Control group, *SD* Standard deviation, *W* Weeks, *M* Months, *TAU* Treatment as usual, *QoL* Quality of Life, *PA* Physical activity, *PROs* Patient-reported outcomes

^a^Significant findings favour the intervention group if not stated otherwise

#### Interventions

Of all 36 included studies, 78% (*n* = 28) focused on lifestyle interventions incorporating both physical activity and eating behaviour [30, 31, 33, 34, 36–43, 47, 48, 50, 51, 53, 55, 57, 60–65], some considering additional risk behaviours such as smoking or substance use [32, 44]. Seven trials (19%) focused only on exercise interventions [35, 46, 49, 52, 56, 58, 59] and one trial only on a dietary intervention [54]. The most common intervention goals were weight management or weight loss, cardiometabolic improvements, and general health promotion. The majority of interventions included psychoeducation, motivational interviewing, and cognitive behavioural strategies such as self-monitoring, goal setting, problem solving, cognitive restructuring, and skills training. Twenty interventions (56%) were group-based [30–32, 35–38, 42, 43, 46, 49, 50, 52–55, 58–60, 64], nine (25%) were a combination of both individual and group elements [40, 44, 45, 47, 51, 56, 57, 62, 65], and seven (19%) were individually targeted [33, 34, 39, 41, 48, 61, 63]. Duration of the interventions ranged from 5 weeks to 12 months, with an average of 26 weeks. All control conditions were nonactive or minimally active.

#### Patient reported outcomes and measures

In the included trials, we found 69 different PROMs. Overall, the most frequently evaluated PROs were (health-related) quality of life, health behaviours, and symptom status. The most frequently used PROMs for QoL were the MOS Short Form Health Surveys SF-36 and the SF-12 [66, 67]. The two most commonly reported health behaviours were physical activity, measured most often with the International Physical Activity Scale (IPAQ) and dietary behaviour measured with food frequency questionnaires, such as the Dietary Instrument for Nutrition Education questionnaire (DINE) [68, 69]. The two most commonly assessed symptoms were depression and anxiety, measured with a variety of PROMs including the Beck Depression Inventory (BDI) and the Symptom Checklist 90 (SCL-90-R) [70, 71]. There was evidence of appropriate psychometric properties of 52% of all PROMs (*n* = 36). Details can be found in the Supplementary Material (Table S2). However, the validity and reliability of 17% of PROMs remained questionable (*n* = 12). This was mostly true for self-reported measures of physical activity and dietary behaviour.

#### Risk of bias

**Fig. 2:**
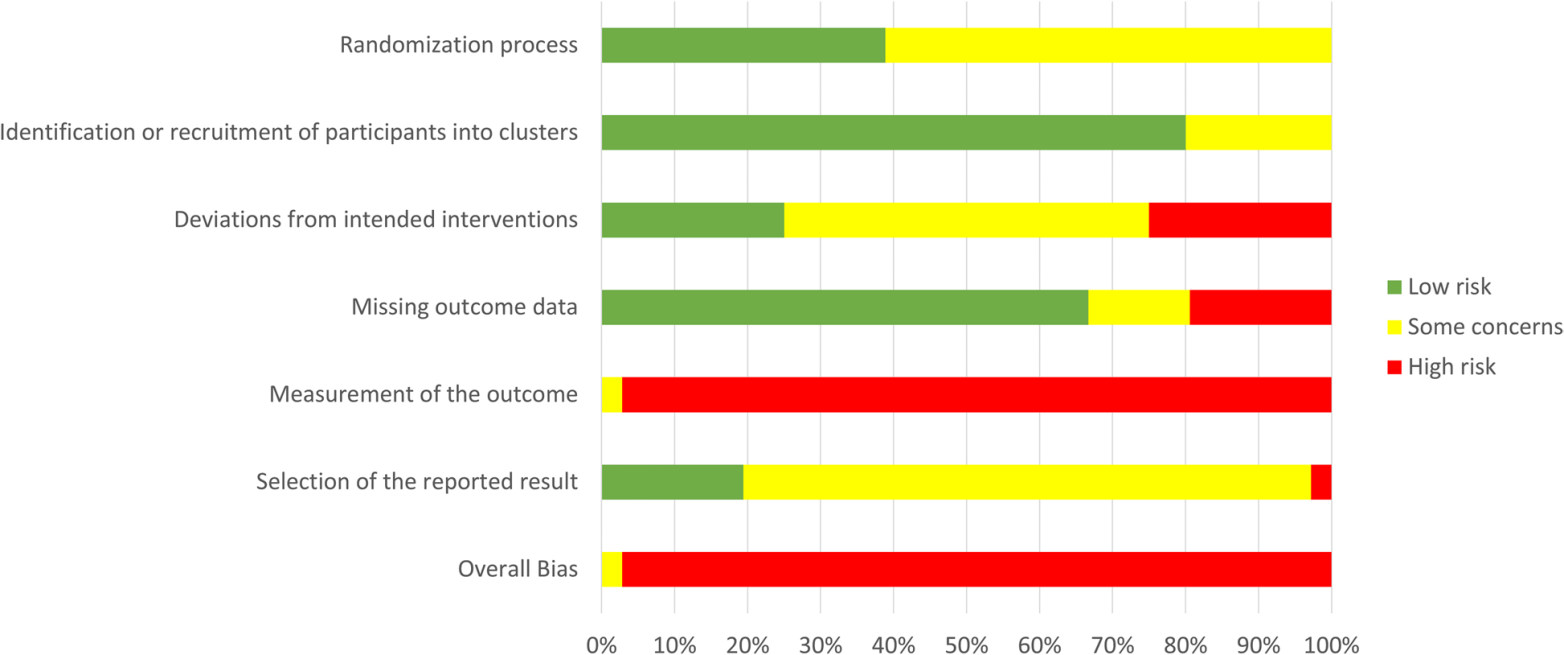
Cochrane risk of bias assessment 2.0

According to the Cochrane risk of bias tool, 35 of the 36 trials were with high risk of bias, and one trial raised some concerns [36] (Fig. 2). Reason for this high risk of bias was the unavoidable lack of blinding of participants and personnel due to the nature of the interventions. When removing that particular domain, 7 of the 36 studies scored a ‘lower risk’ of bias (19%) [32, 34, 44, 47, 48, 53, 62]. The randomisation procedure scored a low risk of bias in 39% of trials (*n* = 14) [32, 34, 37, 44, 47–49, 52, 53, 58, 61, 62, 64, 65]. Few studies (*n* = 11) described allocation concealment [32, 39, 44, 47–49, 52, 58, 62, 64, 65]. Furthermore, 36% of all trials (*n* = 13) seem to have used an appropriate statistical analysis (intention-to-treat without last observation carried forward method) [33, 34, 38, 40, 43–48, 51, 53, 62]. Detailed scores can be found in the Supplementary Material (Fig. S1).

### Results of the meta-analyses

**Fig. 3:**
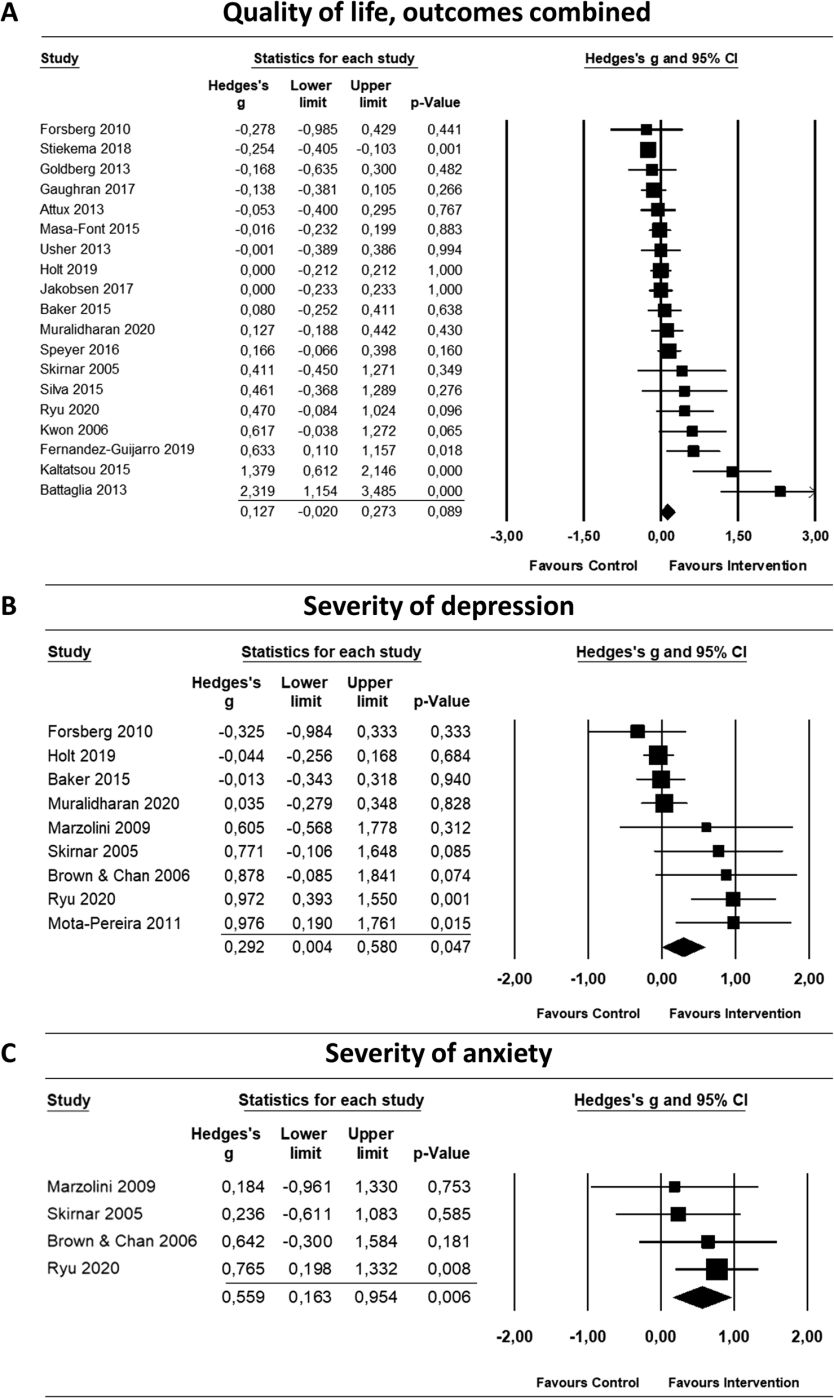
Forest plots of quality of life, depression severity and anxiety severity

We included a total of 21 studies for meta-analysis, some of which included outcomes of more than one analysis. Outcomes of all meta-analyses can be found in Table 2 and forest plots in Fig. 3.

**Table 2:** Meta-analysis and subgroup analysis of the effects of lifestyle interventions for SMI on quality of life, severity of depression and severity of anxiety compared to the control condition

**Post-intervention effect sizes**	***N***	***Hedges' g***	***95% CI***	***I***^***2***^	***p***
Quality of life					
Outcomes combined	19	0.13	(−0.02 to 0.27)	68.7*	0.09
Outcomes combined, outliers removed	17	0.03	(−0.08 to 0.14)	46.0*	0.56
Outcomes with lowest effect size	19	0.10	(−0.05 to 0.24)	67.4*	0.19
Outcomes lowest, outliers removed	17	0.00	(−0.1 to 0.1)	39.6	0.99
Outcomes with highest effect size	19	0.18	(0.02 to 0.33)	73.0*	0.03**
Outcomes highest, outliers removed	17	0.09	(−0.04 to 0.22)	59.8*	0.18
Severity of depression	9	0.292	(0.00 to 0.58)	65.2*	0.047**
Severity of anxiety	4	0.559	(0.16 to 0.95)	0	0.006**
**Subgroup analyses for QoL**	***N***	***Hedges' g***	***95% CI***	***I***^*2*^	***p***
Duration of intervention					
1-3 months	6	0.20	(−0.07 to 0.47)	73.4*	0.05
4-8 months	6	0.37	(0.09 to 0.66)	65.9*	
12 months	7	−0.05	(− 0.25 to 0.15)	49.2	
Region					
Europe	10	0.12	(−0.08 to 0.33)	80.9*	0.94
North America	3	0.07	(−0.34 to 0.49)	0	
Asia/ Pacific	4	0.23	(−0.12 to 0.58)	24.1	
South America	2	0.10	(−0.43 to 0.62)	20.1	
Type of intervention					
Group-based	10	0.30	(−0.07 to 0.53)	73.7*	0.08
Combination	6	−0.07	(−0.29 to 0.16)	37.8	
Individual-based	3	0.18	(−0.16 to 0.51)	40.7	
Attendance to sessions					
High	8	0.46	(0.19 to 0.72)	75.0*	0.01**
Low	8	−0.02	(−0.21 to 0.17)	0	
Unknown	3	0.02	(−0.30 to 0.33)	79.1*	
Risk of bias					
Lower risk of bias	6	−0.06	(− 0.25 to 0.12)	31.9	0.01**
Higher risk of bias	13	0.27	(0.09 to 0.45)	65.7*	
**Explorative analysis (intervention modalities)**	***N***	***Hedges’ g***	***95% CI***	***I***^*2*^	
Mainly structured high intensity PA	5	0.92	(0.31 to 1.53)	65.2*	
Including skill training for healthy diet (i.e. buying groceries, cooking or meal preparation)	4	−0.11	(−0.27 to 0.05)	44.2	
Mainly behavioural therapy components (Motivational interviewing, CBT)	6	0.01	(−0.09 to 0.12)	0	

*N* Number of studies, *CI* confidence interval

^*^p < 0.05, ^**^ statistically significant difference

#### Effects on quality of life

This meta-analysis is based on 19 studies (*n* = 3.129 participants) that evaluated the effect of lifestyle interventions on QoL in patients with SMI. We performed the main analysis calculating combined effect sizes for studies that used more than one outcome measure for QoL. The pooled effect size for quality of life is Hedges’ *g* = 0.13 (95% CI = − 0.02 to 0.27), with a corresponding *p*-value of 0.09, showing no significant increase in QoL in in the intervention groups.

We analysed how the effects would change based on the selection of outcomes with the lower or higher effect size for studies using more than one PROM for QoL. The analysis combining the lowest effect sizes indicated no effect (*g* = 0.1; 95% CI = − 0.05 to 0.24). In contrast, the analysis combining the highest effect sizes indicated a small and statistically significant effect (*g* = 0.18; 95% CI = 0.02 to 0.33; *p* = 0.03).

There was high heterogeneity among QoL studies (*Q* = 57.6, *df* = 18, *p* = 0.00). The null hypothesis of all studies sharing the same common effect size, can be rejected. The *I*^*2*^-statistic is 68.7% (95% CI = 46 to 79), meaning that more than half of the variance in the observed effect reflects the variance of true effects.

#### Effects on depression severity

For the severity of depression, the meta-analysis was based on nine studies (*n* = 790 participants). We found a small significant effect on depression severity with a pooled effect size of *g* = 0.29 (95% CI = 0.00 to 0.58, *p* = 0.047). Heterogeneity appeared to be high among studies evaluating depression severity (*Q* = 23.0, *df* = 8, *p* = 0.003), with an *I*^*2*^ of 65.2% (95% CI = 8 to 81). We did not perform any subgroup analyses on this outcome as the number of studies was too low, yielding a low power of those analyses.

#### Effects on anxiety severity

The meta-analysis on the effects of lifestyle interventions on the severity of anxiety summarized four studies (*n* = 121 participants). We calculated a pooled effect size of *g* = 0.56 (95% CI = 0.16 to 0.95), indicating a moderate and statistically significant effect (*p* = 0.006). The *I*^*2*^-statistic was 0% (95% CI = 0 to 68).

#### Subgroup analysis

For the outcome QoL, five subgroup analyses were performed on the following variables: study region, duration of the intervention, type of intervention, attendance and risk of bias. For the variable attendance, we defined a cut-off value of above 60% for high attendance. For risk of bias, we used the same four domains as for identifying the ‘lower risk’ studies. Risk of bias was significantly associated with the effect size (*p* = 0.01). Studies with a higher risk of bias seemed to show larger effect sizes than those with a lower risk of bias (*g* = 0.27 compared to − 0.06). Furthermore, higher attendance was significantly associated with higher effect sizes (*p* = 0.01), showing an effect size of *g* = 0.46 in the high attendance group compared to − 0.02 in the low attendance group. Studies from the Asian/ Pacific area tended to have a higher effect size compared to other regions (*g* = 0.23; compared to Europe *g* = 0.12, North America *g* = 0.07, and South America *g* = 0.1). Asian studies overlapped to some extend with the ‘higher risk’ of bias studies. Interventions with longer duration (9-12 months) tended to have a lower pooled effect size (*g* = − 0.05, compared to 1-3 months, *g* = 0.2, and 4-8 months *g* = 0.37). In the exploratory analysis we found that interventions including mainly structured high intensity physical activity had a large pooled effect size (*g* = 0.92).

#### Publication bias

The funnel plot of quality of life indicated some of publication bias and Egger’s test of publication bias was significant (*p* = 0.0004). Smaller studies showed more positive results. When imputing missing studies with the trim and fill procedure of Duval and Tweedie, the adjusted effect size was *g* = − 0.05 (95% CI = − 0.12 to 0.017). Funnel plots for depression and anxiety showed no indication for publication bias (Supplementary Material, Fig. S2).

### Grade

The GRADE assessment shows an overall very low quality of the evidence, caused by the high risk of bias, unexplained heterogeneity, and indirectness due to time differences in outcomes (Supplementary Material, Table S3).

### Impact on other patient-reported outcomes

Results for all remaining assessed PRO’s not included in meta-analysis due to the varying outcome concepts and measures showed varying results, overall in favour of lifestyle interventions. An overview of the PRO’s and findings can be found in the descriptive Table 1 and in the Supplementary Material (S2).

Sixteen studies investigated the effects on physical activity [31–34, 36, 37, 39, 41, 42, 44, 45, 48, 53, 55, 58, 61, 63]. Eight of these studies reported improvements in physical activity in the intervention groups in terms of increased minutes of weekly exercise, higher vigorous activity score, and decreased time spent sitting [33, 34, 37, 39, 41, 42, 53, 55].

Sixteen studies evaluated dietary behaviour [31–34, 36, 37, 39, 44, 45, 47, 48, 50, 53–55, 61]. Three studies found significant improvements in the reduction of fat consumption [55], short-term increase of fruit and vegetable consumption [54], and adherence to the Mediterranean diet [36]. Three other studies found significant changes in readiness to change dietary behaviour in favour of the intervention [33, 34, 51].

Eight studies examined smoking behaviour [31, 32, 39, 42, 44, 47, 48, 61] and three studies used PROMs for substance use and alcohol abuse [32, 39, 44]. Neither smoking, alcohol, or substance use were significantly improved by the interventions, except one study in which both groups reduced cigarette consumption [32]. Readiness and motivation to quit smoking or to change health behaviour was assessed by some studies [32, 40, 51], with no significant improvements.

Several studies examined different aspects of perceived mental health [46, 52, 57, 61]. Illness perception and self-appraisal toward illness was assessed and not found improved by two studies [40, 47]. Perceived general health status was assessed by four studies [39, 41, 48, 61], one study showing improvement [41]. Body image and self-esteem were evaluated in four studies [31, 41, 58, 65]. Body image was significantly improved in two of these studies [41, 65]. Weight-related self-esteem was improved in another study [57]. Self-efficacy was measured and found significantly improved in three studies [55, 60, 65]. Sleep quality was found significantly improved in one study [36]. Several studies assessed different aspects of functioning, such as emotional functioning, daily functioning, and independent living skills [31, 43, 46]. One study showed improvements in daily functioning in favour of the intervention group and another in sense of coherence [43, 46].

## Discussion

In this systematic review and meta-analysis, we examined the use of PROs and PROMs in lifestyle intervention trials for people with SMI. We analysed the effect of three PROs that were used in lifestyle intervention trials for people with SMI, namely quality of life, depression and anxiety. We identified 36 studies of which 21 were used for meta-analysis. The most commonly evaluated PROs were quality of life, health behaviours, and symptom status, often reported as secondary or exploratory outcomes. The included studies showed a large variety of different PROMs. The quality the studies was overall low, only seven of the 36 studies had a lower risk of bias.

The meta-analysis showed a very small effect of lifestyle interventions on QoL with an effect size of 0.13, which was not statistically significant (95% CI = − 0.02 to 0.27, *p* = 0.09). The prediction interval for QoL was − 0.41 to 0.66, meaning that the true effect of lifestyle interventions on QoL could be beneficial in some populations and unfavourable in others. In our subgroup analysis were not able to distinguish which patients benefit most from lifestyle interventions, as patient characteristics were too homogeneous. In this respect, also the nature of the lifestyle intervention should be taken into consideration, with the central question which requirements these interventions must meet. The rewarding element for the patient seems to be of great importance. We identified two outlier studies in the meta-analysis of QoL outcomes [35, 49]. Those studies had very large effect sizes, with a Hedges’ *g* = 2.32 (95% CI = 1.15 to 3.49), and *g* = 1.38 (95% CI = 0.61 to 2.51), respectively. Interestingly, those studies used highly social exercise interventions, i.e. soccer practice and Greek traditional dancing. Attendance in these studies was very high. Including these kinds of interactive and social activities in lifestyle interventions could help patients to stay motivated and could increase compliance with, and thus the success of lifestyle interventions. Exploratory analysis revealed high effects for interventions mainly consisting of structured high intensity PA. Although the two outlier studies contributed to this high effect size, the remaining studies likewise showed large effects.

Lifestyle interventions might have the potential to improve mental health outcomes. There were indications of reduction of symptoms of depression and anxiety. The overall effects of lifestyle interventions were small for depression (*g* = 0.29, 95% CI = 0.00 to 0.58, *p* = 0.047) and moderate for anxiety (*g* = 0.56, 95% CI = 0.16 to 0.95, *p* = 0.006). These effect sizes imply a clinically relevant effect [72]. These findings should be confirmed with larger samples. It is also important to note that due to the focus of our review, our findings cannot be generalized to other types of lifestyle interventions, such as smoking cessation or sleep interventions.

Overall, the findings of our meta-analysis are consistent with other systematic reviews. The effect on QoL is similar to the one found in a recent systematic review by Speyer et al., who estimated a nonsignificant SMD of 0.03 (95% CI = − 0.11 to 0.17) in a sample of 15 trials [12]. Our finding on depression severity is in line with a systematic review by Bruins et al. [73]. They found an SMD of − 0.95 (95% CI − 1.90 to − 0.00, *p* = 0.05) reduction on depressive symptoms, which exceeds the effect size that we found. However, Bruins et al. based their results on less studies (*n* = 4). Our findings on depression and anxiety are not reflected in the current meta-review of Firth et al. (2020). Although exercise and healthy diet are protective lifestyle factors for developing depression and anxiety, they do not find significant effects of exercise interventions on depression and anxiety symptoms in persons with schizophrenia [74]. This highlights the issue of implementation errors that could be a possible explanation for the lack of effects. For all kinds of reasons, on the level of the patient or care providers, within the patient-caregiver relationship, or due to team factors, preconditions (e.g. financial or personnel), and other factors, implementation may be less successful, which influences the effectiveness of a lifestyle intervention.

There were considerable differences between the studies in terms of study objectives, methodology, intervention duration, intervention format, and content. This increased the heterogeneity between studies and made it challenging to compare them. We tried to find sources of heterogeneity by analysing different subgroups. Of all subgroup analyses, risk of bias and attendance were significantly associated with the effect sizes. High quality studies led to lower effect sizes, which is also seen in the review of Bruins et al. [73]. This implies that low quality studies tend to overestimate the effects. Our subgroup analysis on attendance showed that studies with higher attendance had significantly higher effects on QoL. A positive correlation of adherence and treatment success was also found in another review [75]. This highlights the importance of patient compliance to maximise treatment effects. Interventions with shorter duration tended to have higher efficacy, which was contrary to our expectations. Speyer et al. and Vancampfort et al. found that studies with an individual approach yield higher effects on weight outcomes [12, 13]. In contrast, other reviews state that group interventions would be more effective and highlight the importance of peer support for motivation [73, 76, 77]. Our own analysis showed a tendency of larger benefits of group settings on QoL. We observed a trend of studies from the Asian region showing larger effect sizes, which is consistent with other systematic reviews [12, 73]. This should be interpreted with caution, as these studies tended to have higher risk of bias. Another possible explanation could be the stricter adherence to interventions in the Asian culture.

### Strengths and limitations

Our systematic review had several strengths. To the best of our knowledge, this paper is the first systematic review and meta-analysis focussing entirely on the evaluation of PROs among lifestyle interventions in patients with SMI. Secondly, we published a predefined study protocol in the beginning of the study period. Thirdly, we conducted a comprehensive and extensive literature search with the support of an expert information specialist, in which no restrictions in terms of language or publication date were applied. However, our search strategy could have included more diet-related search terms. Fourthly, we included only RCTs as these represent the best quality of evidence. On the other hand, despite the inclusion of RCTs only, almost all trials were of a high risk of bias which together with a range of other factors contributed to an overall very low quality of the evidence. Besides that, the lack of power in the meta-analyses of the severity of depression and anxiety weakened the confidence in these results. Study selection was in large parts performed by a single searcher. We tried to limit the possible bias arising the selection procedure by double-screening a sample of 10% of the articles, and by discussing articles of doubt with two or more researchers. Furthermore, we cannot exclude the possibility of missing studies as we excluded non-randomized trials and included published studies only. Unpublished studies could have contributed to a smaller effect, which we tried to simulate in the adjustment of meta-analysis results for QoL by imputing the missing studies. We furthermore cannot exclude the possibility of missing studies in our search, because PROMs are often reported as secondary outcomes or supplementary material. This complicates tracing down these studies in the first phase of study selection while inspecting titles and abstracts. This error could only have been prevented by retrieving the method sections and supplementary materials of eligible studies during the first screening phase. However, we did not believe that this would have been a workable option due to the large number of studies we retrieved.

### Implications for research and clinical practice

Even though lifestyle interventions have modest effects on physical health parameters, there could be other possible benefits that can be captured with PROMs. Despite the value of biomedical outcomes, future trials should involve the patient’s perspective and therefore include PROs to investigate the benefits of lifestyle interventions for SMI in a variety of health concepts. This is particularly critical in mental health research, which often involves outcomes that are difficult or not observable in an objective manner. Researchers should consider PROMs that are matching the aim of their intervention and should choose one measure for every concept that they expect to be influenced by the intervention. The PROM should ideally be valid, reliable, and sensitive to change. Additionally, for the SMI population, the questionnaires should be brief measures that are easy to administer. Self-reported instruments for dietary and exercise tend to be rather inaccurate [78–80]. However, they can still be useful to categorise patients into certain groups and to create awareness of the patient’s health behaviour.

For the clinical setting, the use of more flexible instruments would be advantageous. The National Institutes of Health started the Patient-reported Outcomes Measurement Information system (PROMIS) initiative in order to develop an assessment system for PROs and large item bank which can be used for computerized adaptive testing [81]. This method was shown to provide flexible, efficient, and precise measurements of depression in Dutch patients [82]. Given the promising results, the PROMIS system has the potential to facilitate clinical practice and research in the assessment of PROs.

## Conclusions

The current systematic review and meta-analysis informs mental health professionals on the use of PROs and PROMs in the evaluation of lifestyle intervention trials, and on the effects of lifestyle interventions in patients with SMI on quality of life, depression and anxiety. Despite small and clinically non-significant effects on physical health parameters, lifestyle interventions can however positively affect PROs such as depression and anxiety symptoms, making them more relevant for clinical practice. Comprehensive knowledge of both the clinical and patient-reported outcomes of these programs is necessary in order to choose appropriate treatment for the SMI patient group.

## Supplementary Information

**Additional file 1: Supplementary Table S1.** Full search string PubMed/Medline . **Supplementary Table S2.** List of PROMs in the included studies. **Supplementary Figure S1.** Cochrane risk of bias assessment (detailed). **Supplementary Figure S2.** Publication bias. **Supplementary Table S3.** GRADE summary of evidence.

## Data Availability

All data generated or analysed during this study are included in this published article [and its supplementary information files] and the original studies’ publications.

## Abbreviations

SMI: Severe mental illness
BMI: Body Mass Index
QoL: Quality of life
PRO: Patient-reported outcome
PROM: Patient-reported outcome measure
PRISMA: Preferred Reporting Items for Systematic Reviews and Meta-Analyses
RCT: Randomised controlled trial
GRADE: Grading of Recommendations Assessment, Development, and Evaluation
SMD: Standardized mean difference
