# Supplementary material for: Patient-reported outcomes of lifestyle interventions in patients with severe mental illness: a systematic review and meta-analysis
Source: BMC Psychiatry. 2022 Apr 13;22:261. doi: 10.1186/s12888-022-03854-x (PMC9006587; doi:10.1186/s12888-022-03854-x)
Supplement: Supplementary file 1 — Additional file 1: Supplementary Table S1. Full search string PubMed/Medline . Supplementary Table S2. List of PROMs in the included studies. Supplementary Figure S1. Cochrane risk of bias assessment (detailed). Supplementary Figure S2. Publication bias. Supplementary Table S3. GRADE summary of evidence. [file 12888_2022_3854_MOESM1_ESM.docx]

# Supplementary Material file 1

## Supplementary Table S1 – Full search string PubMed/Medline

| **Search** | **Query** |
| --- | --- |
| #1 | Search: Randomized Controlled Trial[pt] OR "Randomized Controlled Trials as Topic"[Mesh] OR "Random allocation" [Mesh] OR "Double-blind method"[Mesh] OR "Single-blind method"[Mesh] OR random [tw] OR randomly [tw] OR randomised [tw] OR randomized [tw] OR randomising [tw] OR randomizing [tw] OR "Placebos"[Mesh] OR placebo[tiab] OR ((singl*[tw] OR doubl*[tw] OR trebl*[tw] OR tripl*[tw]) AND (mask*[tw] OR blind*[tw] OR dumm*[tw])) OR RCT[tiab] OR RCTs[tiab] OR "Clinical Trials as Topic"[Mesh] OR Clinical Trial [pt] OR clinical trial* [tiab] OR Controlled Clinical Trial [pt] OR controlled trial* [tiab] |
| #2 | Search: "Schizophrenia Spectrum and Other Psychotic Disorders"[Mesh] OR "Personality Disorders"[Mesh] OR "Diagnosis, Dual (Psychiatry)"[Mesh] OR "Bipolar and Related Disorders"[Mesh] OR "Child Development Disorders, Pervasive"[Mesh] OR "Depressive Disorder"[Mesh] OR "Anxiety Disorders"[Mesh] OR "Antipsychotic Agents"[Mesh] OR "SMI"[tiab] OR "severe mental illness*"[tiab] OR "serious mental illness*"[tiab] OR "severe mental disorder*"[tiab] OR "serious mental disorder*"[tiab] OR "dual diagnosis"[tiab] OR "schizophren*"[tiab] OR "psychoti*"[tiab] OR "psychose*"[tiab] OR "psychosis"[tiab] OR "personality disorder*"[tiab] OR "borderline disorder*"[tiab] OR "schizoaffective disorder*"[tiab] OR "schizo-affective disorder*"[tiab] OR "bipolar disorder*"[tiab] OR "manic-depress*"[tiab] OR "mania"[tiab] OR "manias"[tiab] OR "manic state*"[tiab] OR "manic disorder*"[tiab] OR "bipolar depression*"[tiab] OR "euthymic*"[tiab] OR "autistic*"[tiab] OR "autism*"[tiab] OR "pervasive developmental disorder*"[tiab] OR "depressive disorder*"[tiab] OR "major depression"[tiab] OR "anxiety disorder*"[tiab] OR "antipsychotic*"[tiab] OR "antidepressants"[tiab] OR "mood stabilizers"[tiab] |
| #3 | Search: "Life Style"[Mesh] OR "Physical Fitness"[Mesh] OR "Exercise"[Mesh] OR "Health Promotion"[Mesh] OR "Health Behavior"[Mesh:NoExp] OR "life style*"[tiab] OR "lifestyle*"[tiab] OR "intensive lifestyle intervention"[tiab] OR "lifestyle modification"[tiab] OR "lifestyle change"[tiab] OR "weight loss program"[tiab] OR "weight reduction program"[tiab] OR "obesity"[tiab] OR "obese"[tiab] OR "abdominal obesity"[tiab] OR "weight management"[tiab] OR "BMI"[tiab] OR "body mass index"[tiab] OR "weight"[tiab] OR "overweight"[tiab] OR "weight gain"[tiab] OR "weight change"[tiab] OR "weight loss"[tiab] OR "body weight"[tiab] OR "exercise*"[tiab] OR "physical activit*"[tiab] OR "physical fitness"[tiab] OR "fitness"[tiab] OR "healthy diet"[tiab] OR "healthy lifestyle"[tiab] OR "health promotion"[tiab] OR "healthy living"[tiab] OR "physical inactivit*"[tiab] OR "prolonged sitting"[tiab] OR "stationary behavio*"[tiab] OR "sedentary behavior*"[tiab] OR "sedent*"[tiab] |
| #4 | Search: "Patient Reported Outcome Measures"[Mesh] OR "Quality of Life"[Mesh] OR "prom"[tiab] OR "proms"[tiab] OR "pro"[tiab] OR "pros"[tiab] OR "HRQL"[tiab] OR "HRQoL"[tiab] OR "QL"[tiab] OR "QoL"[tiab] OR "quality of life"[tiab] OR "life quality"[tiab] OR "health index*"[tiab] OR "health indices"[tiab] OR "health profile*"[tiab] OR "health status"[tiab] OR ((patient[tiab] OR self[tiab]) AND ((report[tiab] OR reported[tiab] OR reporting[tiab]) OR (rated[tiab] OR rating[tiab] OR ratings[tiab]) OR based[tiab] OR (assessed[tiab] OR assessment[tiab] OR assessments[tiab]))) OR ((disability[tiab] OR function[tiab] OR functional[tiab] OR functions[tiab] OR subjective[tiab] OR utility[tiab] OR utilities[tiab] OR wellbeing[tiab] OR well being[tiab]) AND (outcome[tiab] OR outcomes[tiab] OR index[tiab] OR indices[tiab] OR instrument[tiab] OR instruments[tiab] OR measure[tiab] OR measures[tiab] OR questionnaire[tiab] OR questionnaires[tiab] OR profile[tiab] OR profiles[tiab] OR scale[tiab] OR scales[tiab] OR score[tiab] OR scores[tiab] OR status[tiab] OR survey[tiab] OR surveys[tiab])) OR "Patient-reported outcome"[tiab] OR "self-reported outcome"[tiab] OR "person reported outcome"[tiab] OR "wellbeing"[tiab] OR "self-perceived health"[tiab] OR "self-rated health"[tiab] OR "health belief*"[tiab] OR "global functioning"[tiab] OR "physical functioning"[tiab] OR "mental functioning"[tiab] OR "social functioning"[tiab] OR "occupational functioning"[tiab] OR "vocational functioning"[tiab] OR "cognitive functioning"[tiab] OR "functional recovery"[tiab] OR "patient centered outcome*"[tiab] OR "patient outcome measure*"[tiab] OR "patient outcome scale*"[tiab] OR "Self-rated"[tiab] OR "self-efficacy"[tiab] OR "self-esteem"[tiab] OR "self-management"[tiab] OR "self-reported"[tiab] OR "wellness"[tiab] OR "symptom severity"[tiab] OR "exploratory outcome*"[tiab] Sort by: Most Recent |
| #5 | #1 AND #2 AND #3 AND #4 |

## Supplementary Table S2 – List of PROMs in the included studies

| **PRO** | **PROM** |
| --- | --- |
| Quality of life (QoL) | MOS Short Form Health Survey (SF-36) (1)  Manchester Short Assessment of Quality of Life (MANSA) (2)  MOS Short Form Health Survey (SF-12) (3)  World Health Organisation Quality of life (WHO-QOL-BREF) (4)  Impact of Weight on Quality of Life (IWOQOL) (5)  EuroQoL (EQ-5D) (6)  Q-LES-Q (Quality of Life Enjoyment and Satisfaction Questionnaire) (7)  Lehman Quality of Life Questionnaire (8)  Veterans RAND-12 (9) |
| Symptom status | Beck Depression Inventory (BDI) (10)  Patient Health Questionnaire (PHQ-9) (11)  Hospital Anxiety and Depression Scale (HAD) (12)  Symptom Checklist 90 (SCL-90-R) (13)  State-Trait Anxiety Inventory (STAI) (14)  Revised Behaviour and Symptom Identification Scale (BASIS-R) (15)  Drug Attitude Inquiry (DAI-10) (16)  Liverpool University Neuroleptic Side Effect Rating Scale (LUNSERS) (17)  Perceived Stress Scale (PSS) (18) |
| Functional status | Independent Living Skills Survey (ILSS) (19)  Barthel's activity of daily living (ADL) (20)  Lawton's Instrumental Activities of Daily Living Scale (IADL) (21)  Sense of coherence scale (SOC) (22)  Pittsburgh Sleep quality (PSQI) (23) |
| General Health Perception | Brief Illness Perception Questionnaire (Brief-IPQ) (24)  Self-Appraisal of Illness Questionnaire (SAIQ) (25)  Mental Health Inventory (MHI) (26) |
| Health behaviours | International Physical Activity Questionnaire (IPAQ) (27)  Physical Activity Scale (PAS) (28)  Yale Physical Activity Scale (YPA) (29)  Godin-Shephard Leisure-Time Exercise Questionnaire (LTEQ) (30)  Physical Activity Scale for the Elderly - Korean Version (K-PASE) (31)  Dietary Instrument for Nutrition Education (DINE) (32)  Brief Block Food Frequency Questionnaire (FFQ) (33)  Mediterranean Diet Assessment Tool (34)  Dietary Quality Score (DQS) (35)  Fagerström Nicotine Dependence Questionnaire (36)  Fagerström Tolerance Questionnaire (36)  Opiate Treatment Index (OTI) (37)  Alcohol Use Disorders Identification Test (AUDIT) (38)  Time line follow back (TLFB) (39) |
| Other | Client Satisfaction Questionnaire (CSQ-8) (40)  Three-item-loneliness Scale (41)  Diabetes Empowerment Scale (DAS) (42)  Patient Activation Measure (PAM) (43)  Boston University Empowerment Scale (BUES) (44)  Rosenberg Self-esteem Scale (RSES) (45)  Body Weight, Image and Self-Esteem (B-WISE) (46)  Readiness and Motivation to Quit Smoking (RMQ) (47) |

## Supplementary Figure S1 – Cochrane risk of bias assessment (detailed)

## Supplementary Figure S2 – Publication bias
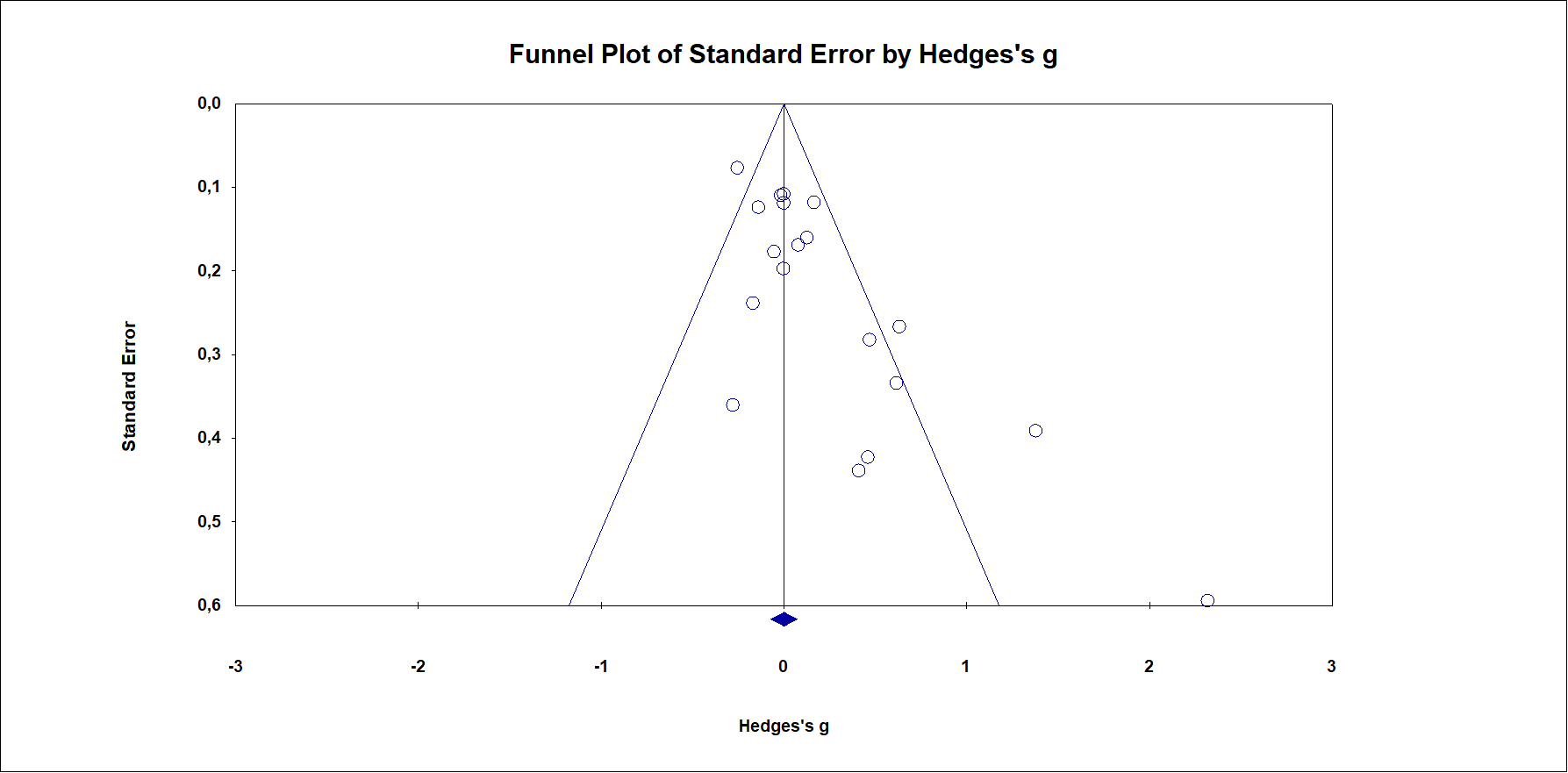


S2a) Funnel plot for quality of life (outcomes combined)


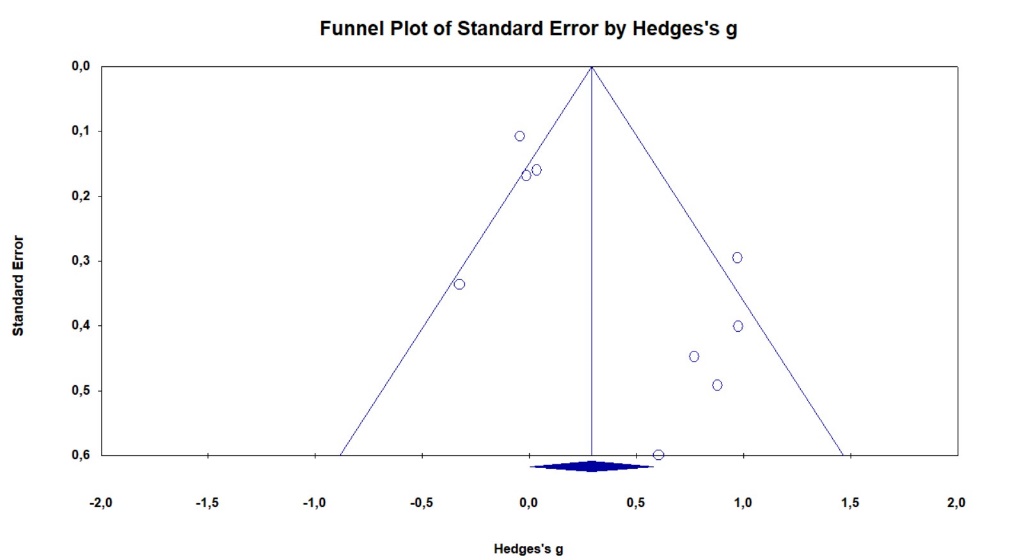


S2b) Funnel plot for severity of depression


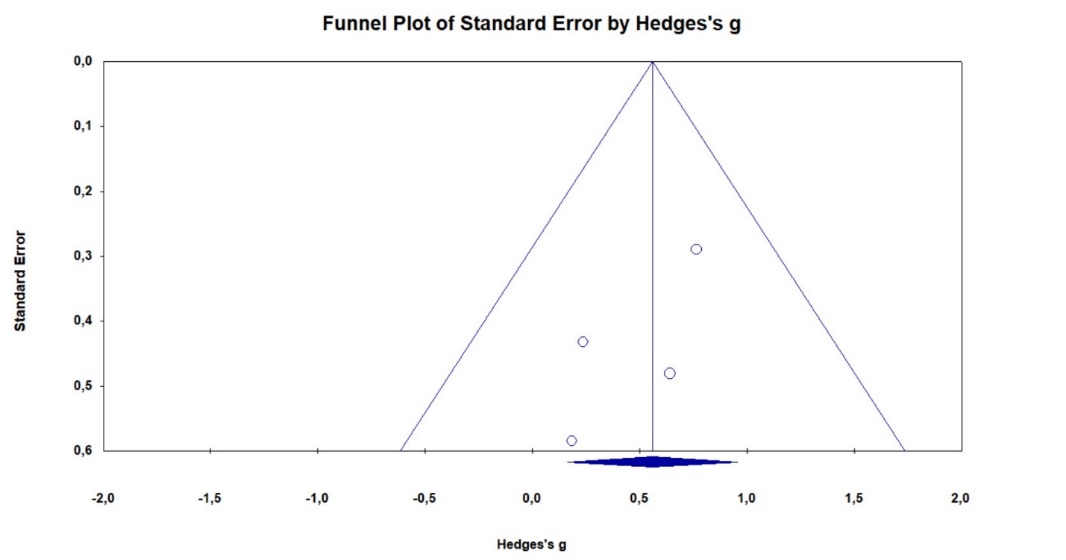


S2c) Funnel plot for severity of anxiety

## Supplementary Table S3 – GRADE summary of evidence

| **Lifestyle interventions compared to control condition for patients with severe mental illness** | | | | | | |
| --- | --- | --- | --- | --- | --- | --- |
| **Patient or population:** patients with severe mental illness  **Setting:** Outpatient, supported housing, or inpatient  **Intervention:** Lifestyle interventions  **Comparison:** control condition | | | | | | |
| Outcomes | **Anticipated absolute effects^*^** (95% CI) | | Relative effect (95% CI) | № of participants (studies) | Certainty of the evidence (GRADE) | Comments |
|  | **Risk with control condition** | **Risk with Lifestyle interventions** |  |  |  |  |
| Quality of life (QoL) assessed with: Various PROMs follow-up: range 12 weeks to 12 months | The mean quality of life was **0** units | Hedges's g **0.127 units higher** (0.02 lower to 0.273 higher) | - | 3129 (19 RCTs) | ⨁◯◯◯ Very low |  |
| Depression severity assessed with: Various PROMs follow-up: range 6 weeks to 12 months | The mean depression severity was **0** units | Hedges's g **0.292 units higher** (0.004 higher to 0.58 higher) | - | 790 (9 RCTs) | ⨁◯◯◯ Very low |  |
| Anxiety severity assessed with: Various PROMs follow-up: range 6 weeks to 16 weeks | The mean anxiety severity was **0** units | Hedges's g **0.559 units higher** (0.163 higher to 0.954 higher) | - | 121 (4 RCTs) | ⨁◯◯◯ Very low |  |
| ***The risk in the intervention group** (and its 95% confidence interval) is based on the assumed risk in the comparison group and the **relative effect** of the intervention (and its 95% CI).  **CI:** confidence interval | | | | | | |
| **GRADE Working Group grades of evidence** **High certainty:** we are very confident that the true effect lies close to that of the estimate of the effect. **Moderate certainty:** we are moderately confident in the effect estimate: the true effect is likely to be close to the estimate of the effect, but there is a possibility that it is substantially different. **Low certainty:** our confidence in the effect estimate is limited: the true effect may be substantially different from the estimate of the effect. **Very low certainty:** we have very little confidence in the effect estimate: the true effect is likely to be substantially different from the estimate of effect. | | | | | | |

## References

1. Su C-T, Ng H-S, Yang A-L, Lin C-Y. Psychometric evaluation of the Short Form 36 Health Survey (SF-36) and the World Health Organization Quality of Life Scale Brief Version (WHOQOL-BREF) for patients with schizophrenia. Psychological Assessment. 2014;26(3):980-9.

2. Priebe S, Huxley P, Knight S, Evans S. Application and Results of the Manchester Short Assessment of Quality of Life (Mansa). International Journal of Social Psychiatry. 1999;45(1):7-12.

3. Ware JE, Kosinski M, Keller SD. A 12-Item Short-Form Health Survey: Construction of Scales and Preliminary Tests of Reliability and Validity. Medical Care. 1996;34(3):220-33.

4. Kolotkin RL, Crosby RD, Kosloski KD, Williams GR. Development of a Brief Measure to Assess Quality of Life in Obesity. Obesity Research. 2001;9(2):102-11.

5. Brazier J, Jones N, Kind P. Testing the validity of the Euroqol and comparing it with the SF-36 health survey questionnaire. Quality of Life Research. 1993;2(3):169-80.

6. König H-H, Roick C, Angermeyer MC. Validity of the EQ-5D in assessing and valuing health status in patients with schizophrenic, schizotypal or delusional disorders. European Psychiatry. 2007;22(3):177-87.

7. Wyrwich K, Harnam N, Revicki DA, Locklear JC, Svedsäter H, Endicott J. Assessing health-related quality of life in generalized anxiety disorder using the Quality Of Life Enjoyment and Satisfaction Questionnaire. Int Clin Psychopharmacol. 2009;24(6):289-95.

8. Nørholm V, Bech P. Quality of life assessment in schizophrenia: Applicability of the Lehman Quality of Life Questionnaire (TL-30). Nordic Journal of Psychiatry. 2007;61(6):438-42.

9. Kazis LE, Miller DR, Clark JA, Skinner KM, Lee A, Ren XS, et al. Improving the response choices on the veterans SF-36 health survey role functioning scales: results from the Veterans Health Study. J Ambul Care Manage. 2004;27(3):263-80.

10. Beck AT, Steer RA, Carbin MG. Psychometric properties of the Beck Depression Inventory: Twenty-five years of evaluation. Clinical Psychology Review. 1988;8(1):77-100.

11. Kroenke K, Spitzer RL, Williams JBW. The PHQ-9. Journal of General Internal Medicine. 2001;16(9):606-13.

12. Bjelland I, Dahl AA, Haug TT, Neckelmann D. The validity of the Hospital Anxiety and Depression Scale. An updated literature review. J Psychosom Res. 2002;52(2):69-77.

13. Schmitz N, Hartkamp N, Kiuse J, Franke GH, Reister G, Tress W. The Symptom Check-List-90-R (SCL-90-R): A German validation study. Quality of Life Research. 2000;9(2):185-93.

14. Balsamo M, Romanelli R, Innamorati M, Ciccarese G, Carlucci L, Saggino A. The state-trait anxiety inventory: shadows and lights on its construct validity. Journal of Psychopathology and Behavioral Assessment. 2013;35(4):475-86.

15. Susan VE, Normand S-L, Belanger AJ, Avron S, David E. The Revised Behavior and Symptom Identification Scale (BASIS-R): Reliability and Validity. Medical Care. 2004;42(12):1230-41.

16. Nielsen RE, Lindström E, Nielsen J, Levander S. DAI-10 is as good as DAI-30 in schizophrenia. European Neuropsychopharmacology. 2012;22(10):747-50.

17. Day JC, Wood G, Dewey M, Bentall RP. A Self-Rating Scale for Measuring Neuroleptic Side-Effects. British Journal of Psychiatry. 1995;166(5):650-3.

18. Cohen S, Kamarck T, Mermelstein R. A global measure of perceived stress. Journal of Health and Social Behavior. 1983;24(4):385-96.

19. Wallace CJ, Liberman RP, Tauber R, Wallace J. The Independent Living Skills Survey: A Comprehensive Measure of the Community Functioning of Severely and Persistently Mentally Ill Individuals. Schizophrenia Bulletin. 2000;26(3):631-58.

20. Leung SOC, Chan CCH, Shah S. Development of a Chinese version of the Modified Barthel Index — validity and reliability. Clinical Rehabilitation. 2007;21(10):912-22.

21. Tong AYC, Man DWK. The Validation of the Hong Kong Chinese Version of the Lawton Instrumental Activities of Daily Living Scale for Institutionalized Elderly Persons. OTJR: Occupation, Participation and Health. 2002;22(4):132-42.

22. Eriksson M. Validity of Antonovsky's sense of coherence scale: a systematic review. Journal of Epidemiology & Community Health. 2005;59(6):460-6.

23. Curcio G, Tempesta D, Scarlata S, Marzano C, Moroni F, Rossini PM, et al. Validity of the Italian Version of the Pittsburgh Sleep Quality Index (PSQI). Neurological Sciences. 2013;34(4):511-9.

24. Broadbent E, Petrie KJ, Main J, Weinman J. The Brief Illness Perception Questionnaire. Journal of Psychosomatic Research. 2006;60(6):631-7.

25. Marks KA, Fastenau PS, Lysaker PH, Bond GR. Self-Appraisal of Illness Questionnaire (SAIQ): relationship to researcher-rated insight and neuropsychological function in schizophrenia. Schizophrenia Research. 2000;45(3):203-11.

26. Cuijpers P, Smits N, Donker T, Ten Have M, De Graaf R. Screening for mood and anxiety disorders with the five-item, the three-item, and the two-item Mental Health Inventory. Psychiatry Research. 2009;168(3):250-5.

27. Craig CL, Marshall AL, Sjöström M, Bauman AE, Booth ML, Ainsworth BE, et al. International Physical Activity Questionnaire: 12-Country Reliability and Validity. Medicine & Science in Sports & Exercise. 2003;35(8):1381-95.

28. Andersen LG, Groenvold M, Jørgensen T, Aadahl M. Construct validity of a revised Physical Activity Scale and testing by cognitive interviewing. Scandinavian Journal of Public Health. 2010;38(7):707-14.

29. Abajo S, Larriba R, Márquez S. Validity and reliability of the Yale Physical Activity Survey in Spanish elderly. The Journal of sports medicine and physical fitness. 2002;41:479-85.

30. Amireault S, Godin G. The Godin-Shephard Leisure-Time Physical Activity Questionnaire: Validity Evidence Supporting its Use for Classifying Healthy Adults into Active and Insufficiently Active Categories. Perceptual and Motor Skills. 2015;120(2):604-22.

31. Washburn RA, Smith KW, Jette AM, Janney CA. The physical activity scale for the elderly (PASE): Development and evaluation. Journal of Clinical Epidemiology. 1993;46(2):153-62.

32. Roe L, Strong C, Whiteside C, Neil A, Mant D. Dietary intervention in primary care: validity of the DINE method for diet assessment. Fam Pract. 1994;11(4):375-81.

33. Subar AF, Thompson FE, Kipnis V, Midthune D, Hurwitz P, McNutt S, et al. Comparative Validation of the Block, Willett, and National Cancer Institute Food Frequency Questionnaires. American Journal of Epidemiology. 2001;154(12):1089-99.

34. Martínez-González MA, García-Arellano A, Toledo E, Salas-Salvadó J, Buil-Cosiales P, Corella D, et al. A 14-Item Mediterranean Diet Assessment Tool and Obesity Indexes among High-Risk Subjects: The PREDIMED Trial. PLoS ONE. 2012;7(8):e43134.

35. Toft U, Kristoffersen LH, Lau C, Borch-Johnsen K, Jørgensen T. The Dietary Quality Score: validation and association with cardiovascular risk factors: the Inter99 study. European Journal of Clinical Nutrition. 2007;61(2):270-8.

36. Heatherton TF, Kozlowski LT, Frecker RC, Fagerström KO. The Fagerström Test for Nicotine Dependence: a revision of the Fagerström Tolerance Questionnaire. Br J Addict. 1991;86(9):1119-27.

37. Darke S, Hall W, Wodak A, Heather N, Ward J. Development and validation of a multi-dimensional instrument for assessing outcome of treatment among opiate users: the Opiate Treatment Index. Br J Addict. 1992;87(5):733-42.

38. Daeppen J-B, Yersin B, Landry U, Pecoud A, Decrey H. Reliability and Validity of the Alcohol Use Disorders Identification Test (AUDIT) Imbedded Within a General Health Risk Screening Questionnaire: Results of a Survey in 332 Primary Care Patients. Alcoholism: Clinical and Experimental Research. 2000;24(5):659-65.

39. Hjorthøj CR, Hjorthøj AR, Nordentoft M. Validity of Timeline Follow-Back for self-reported use of cannabis and other illicit substances — Systematic review and meta-analysis. Addictive Behaviors. 2012;37(3):225-33.

40. Attkisson CC, Zwick R. The client satisfaction questionnaire. Evaluation and Program Planning. 1982;5(3):233-7.

41. Hughes ME, Waite LJ, Hawkley LC, Cacioppo JT. A Short Scale for Measuring Loneliness in Large Surveys. Research on Aging. 2004;26(6):655-72.

42. Anderson RM, Funnell MM, Fitzgerald JT, Marrero DG. The Diabetes Empowerment Scale: a measure of psychosocial self-efficacy. Diabetes Care. 2000;23(6):739-43.

43. Hibbard JH, Stockard J, Mahoney ER, Tusler M. Development of the Patient Activation Measure (PAM): Conceptualizing and Measuring Activation in Patients and Consumers. Health Services Research. 2004;39(4p1):1005-26.

44. Rogers ES, Chamberlin J, Ellison ML, Crean T. A consumer-constructed scale to measure empowerment among users of mental health services. Psychiatric Services. 1997;48(8):1042-7.

45. Rosenberg M. Rosenberg self-esteem scale (RSE). Acceptance and commitment therapy Measures package. 1965;61(52):18.

46. Awad AG, Voruganti LN. Body weight, image and self-esteem evaluation questionnaire: development and validation of a new scale. Schizophr Res. 2004;70(1):63-7.

47. Crittenden KS, Manfredi C, Warnecke RB, Cho YI, Parsons JA. Measuring readiness and motivation to quit smoking among women in public health clinics: predictive validity. Addictive Behaviors. 1998;23(2):191-9.
